# Participatory Research to Design a Novel Telehealth System to Support the Night-Time Needs of People with Dementia: NOCTURNAL

**DOI:** 10.3390/ijerph10126764

**Published:** 2013-12-04

**Authors:** Suzanne Martin, Juan Carlos Augusto, Paul Mc Cullagh, William Carswell, Huiru Zheng, Haiying Wang, Jonathan Wallace, Maurice Mulvenna

**Affiliations:** 1 Faculty of Life and Health Sciences, University of Ulster, Northern Ireland BT37 0QB, UK; 2 School of Science and Technology, Middlesex University London, England NW4 4BT, UK; E-Mail: j.augusto@mdx.ac.uk; 3 Faculty of Computing and Mathematics, University of Ulster, Northern Ireland BT37 0QB, UK; E-Mails: pj.mccullagh@ulster.ac.uk (P.M.C.); w.carswell@ulster.ac.uk (W.C.); h.zheng@ulster.ac.uk (H.Z.); hy.wang@ulster.ac.uk (H.W.); jg.wallace@ulster.ac.uk (J.W.); md.mulvenna@ulster.ac.uk (M.M.)

**Keywords:** dementia, ambient assisted living, participatory design

## Abstract

Strategies to support people living with dementia are broad in scope, proposing both pharmacological and non-pharmacological interventions as part of the care pathway. Assistive technologies form part of this offering as both stand-alone devices to support particular tasks and the more complex offering of the “smart home” to underpin ambient assisted living. This paper presents a technology-based system, which expands on the smart home architecture, orientated to support people with daily living. The system, NOCTURNAL, was developed by working directly with people who had dementia, and their carers using qualitative research methods. The research focused primarily on the nighttime needs of people living with dementia in real home settings. Eight people with dementia had the final prototype system installed for a three month evaluation at home. Disturbed sleep patterns, night-time wandering were a focus of this research not only in terms of detection by commercially available technology but also exploring if automated music, light and visual personalized photographs would be soothing to participants during the hours of darkness. The NOCTURNAL platform and associated services was informed by strong user engagement of people with dementia and the service providers who care for them. NOCTURNAL emerged as a holistic service offering a personalised therapeutic aspect with interactive capabilities.

## 1. Introduction

The challenges of an ageing population within which people are living longer with congenital and chronic disease are well documented. The particular difficulties however presented by Alzheimer’s disease or other dementias create unique and individual problems for the person living with the condition and their family and friends. Whilst Alzheimer’s disease is more prevalent in the older population, it is also evident in other groups, for example people who have experienced head trauma or traumatic brain injury [1] and those individuals born with Downs Syndrome often present with early onset [2] and have an increased risk of developing the condition. Dementia is a progressive syndrome in which there is deterioration in cognitive function beyond what is experienced during normal ageing. Dementia is an increasingly common condition among older people; affecting over 820,000 people in the United Kingdom (UK) [3] and almost 35.6 million people worldwide [4]. There are many different types of dementia, with Alzheimer’s disease being the most common [5]. The symptoms vary between individuals and according to the type of dementia and severity of the syndrome. Memory deficits are one of the most common characteristics of dementia along with cognitive decline. This often results in the person living with dementia having problems generating coherent speech or understanding the spoken or written word. They may have difficulty identifying previously familiar objects, exhibit behavioural changes, decreased judgement and difficulties with activities of daily living. Furthermore, disorientation and wandering may be an emerging feature as the condition deteriorates [6,7]. The Alzheimer’s association (2012) estimate that 1 in 8 older Americans or 5.4 million of the total population of the USA has Alzheimer’s disease. This figure includes 5.2 million people age 65 and older and 200,000 individuals under age 65 who have younger-onset Alzheimer’s [7].

For people with Alzheimer’s disease and other dementias, aggregate payments for health care, long-term and hospice care are projected to increase in the USA from $183 billion in 2011 to $1.1 trillion in 2050. The current financial cost of dementia care in the UK is estimated to be £23 billion and this is expected to rise to £27 billion by 2018 [8]. 

Sleep disturbances often present within dementia, being characterised by “sundowning” and sleep apnoea, alongside disturbances of movement control. Sundowning, whilst poorly defined as a phenomenon, may present as the recurring onset of confusion or agitation in the person with dementia during the evening. Irregular sleep-wake pattern is most common in cognitively impaired people, with irregular sleep-wake patterns consisting of temporally disorganized and irregular sleep and waking behaviour [9,10]. Dewing found that people with dementia can experience “40 per cent of their bedtime hours awake and 14 per cent of their daytime hours asleep” [11]. Kerr suggests that the primary emphasis in nighttime care is on supporting the person in the activity of sleep and this could become the only objective during the night-time period. While sleep has a positive physiological restorative function she proposes that the night can also be a time when other beneficial activities can be carried out with people who awaken [12]. 

It is very often the risks, real or perceived, during the hours of darkness that will cause an admission into institutional care. The findings of the SOMNIA project [13] conclude that as people age they waken not because of worry but rather pain. The team designed and evaluated four technologies for unsupervised use in care homes. These included a nighttime tray, a musical pillow, automatic lighting and a nighttime communicator which was hand held to the ear for those people who used a hearing aid during the day. In addition staff informed the team that information on a person’s breathing and enuresis would be helpful to ensure they can meet individual care needs without disturbing other staff. The team then explored how commercially available sensors for vital signs, urine detection, movement, *etc*. could be combined to provide sufficient remote indication of the resident’s status that staff would not feel they needed to disturb the resident at night. Orpwood *et al*. adopted a single case study design to evaluate the impact of a smart home installation on the behavior and independence of someone with severe dementia. This research found that technology enabled the client to retain a lot of independence. It helped him to regain urinary continence, improved his sleep from around 3.5 h per night to 5.5, and halved the number of night-time wanderings [14]

Many people with dementia eventually have to leave their own home and live in institutionalized care, as the need for health and social care support increases. To address this personal catastrophe and lack of societal support many interventions have been proposed. Interventions include pharmacological and non- pharmacological options ranging from cognitive stimulation therapy [15], acupuncture [16] reminiscence [17] to music therapy [18]. National governments have developed “dementia” strategies, and public healthcare agencies have set down best practice guidelines for care [19]. 

There is a plethora of literature on technology to support autonomous living, participation, and enable self-management of chronic diseases. The domains for publication output are multifaceted, which reflects the inherent breadth of interest and contributions required to achieve advancement in this multi-modal, multi-dimensional research area. However, whilst the availability of empirical research evidence is somewhat lacking [20] there is significant knowledge to be gained from scoping best practice and developments. Flemming and Shima reviewing the literature concluded that technology was often found to be unreliable, and there was a ‘marked resistance’ to the acceptance of most available technologies [21]. Furthermore they stated that technology should be tailored to the needs of the individual. 

Carswell *et al*. focused their literature review specifically on technology to support people with dementia during the hour of darkness. This review highlighted a need for more research that focuses on the unique nighttime needs of people with dementia living in their own home. In addition the authors postulated that there is the potential to use technology as a therapeutic intervention in addition to the more traditional applications [22]. In addition Kujala undertook a review exploring the evidence base for user involvement in technology design. The key findings of this work were low but reliable correlations between user participation and overall system acceptance, acceptance of the system functionality, enhanced system usage and overall user satisfaction [23].

The literature revealed a gap in research that directly involved people with dementia, in addition to the opportunity to develop a comprehensive technical solution that not only managed risk but also provided a therapeutic intervention. Thus this study adopted a qualitative research methodology with a participatory design involving people with dementia and their carers. The lead investigator working directly with users is influenced by descriptive phenomenology [24] as she sought to describe the lived experience of those participants with dementia and their carers within this research project. The methods combined qualitative observations, adapted interviews [25] and technical analysis of system usage by participants. The research was carried out in real home environments; addressing the fitness of current smart home technologies otherwise known as Ambient Assisted Living (AAL) solutions for real world deployment. Aiming to focus specifically on the nighttime needs of people with dementia, extending the potential of the ambient assisted living home to create a personalized component that would deliver a therapeutic dimension. 

There are many commercially available stand-alone devices or integrated sensor solutions with applications to support people with dementia at home [26,27]. The aim of this study was to explore if technology could provide pervasive ambient assisted living to support people with dementia at home and move beyond this to offer therapeutic interventions for example of reminiscence and music. The purpose and proposed novelty of NOCTURNAL was moving beyond risk management and telecare to enable a therapeutic component to the platform. This would include reality orientation, and personalized reminisce tools which could be used manually by the person with dementia and would be automated to initiate during the night in response to sensor activity which correlated to disturbed sleep. 

## 2. Methodology

The research approach is grounded in the philosophy and practices of the “Translating Research and Innovation Living Lab” methodology [28,29,30]. User participation of people with dementia, their carers and service providers was part of the methods. Best practice in dementia research strongly recommends involving those people living with the condition [31,32,33]. The methods of Allen [34] and Dewing [35] of adopting a “process consent method” to support engagement, was adopted by the team. In this approach “permission” was sought to engage with the person with dementia, understanding how they normally give consent for activities *etc*. and uses this to inform decision making around daily/weekly consent monitoring. Methods to support engagement and seek consent for each individual were explored with those who cared for the people with dementia [36]. A secondary aim of the project was achieving commercialization readiness for the final service. The project consortium included a cross faculty academic team (bringing together Health Sciences and Computing and Mathematics) alongside FOLD telecare [37]; the major commercial provider of social alarms, telecare and telehealth on the island of Ireland. In addition to our formal partners, research collaborators included two public sector Health and Social Care (HSC) providers *i.e.*, the Northern Trust and Southeastern Trust, and PRAXIS [38] who are a non-government agency providing of housing and care for people with dementia. 

The HSC provided a purposive sample of the target population. Inclusion criteria were; people with early stage dementia with a confirmed diagnosis of dementia classified as stage 4; moderate cognitive decline (mild or early stage Alzheimer’s disease). The individual also had to be in receipt of a care package and living in his or her own home, or holding tenure in support living. 

A qualitative research study design was developed to ensure that a strong participatory approach informed all stages of the work. The project framework included a variety of work packages dealing with the specific tasks of:

1. Gathering user requirements using a user centred design approach.
2. System development and integration.
3. Data analysis and decision support.
4. Trial evaluation, analysis and validation.

The process adopted to support engagement with the participants was a follows:

1. A referral was sent from HSC to FOLD, a project partner.
2. An email was sent to the Lead on user engagement (SM) and copied to the PI.
3. SM would then contact all those on the referral to set up an initial face to face visit. This initial visit would last no more than 30 min, during which time project would be discussed in detail with potential participants and carers or invited significant other of their choice. The concept of NOCTURNAL and requirement from each participant will be clearly explained and SM would regularly check the understanding of those in attendance during the meeting. A participant information sheet for each person attending was provided. The issue of consent to participate and also how to withdraw from the project outlined. The consent to participate form was ‘walked’ through, and left with those present to reflect upon. A date for a follow-up visit was agreed for one week’s time to discuss the project and seek consent to participate.
4. The second meeting one week later would also last no more than 30 min and was led by SM. She recapped on the project and sought views on consent to participate. If consent was forthcoming SM would begin the process of starting to gather user information. Based on semi-structure interviews with a phenomenological approach SM would probe on the participants lived experience for example:
  - Describe a good night at home
  - Tell me what helps you feel safe at home during the hours of darkness?
  - How do you feel at night?
  - Do you feel satisfied with your night-time routine?
  - What do you understand by technology?
  - When you think about technology how does it make you feel?
  - What challenges do you think that using technology would present to you?
  - Can you see any benefits of using technology?

The aim was to establish the main issues/risks and care needs that arise during the hours of darkness.

Ethical approval was given to tape and transcribe these interviews. These were then thematically analysis producing emergent broad topics which included:

- Promoting independence
- Maintaining dignity
- Maximising social inclusion
- Managing risk
- Providing stimulation

Once gathering user requirements was complete the participants were then involved in system validation and evaluation. There were three phases of this planned within the project, each planned to last for 3 months. These are broadly outlined below:

*Phase one:* this was validation and evaluation of first stage prototype of NOCTURNAL. At this stage the key aim was to test the stability of the system as we moved from the lab to the home and integrated from home to FOLD. During this stage prototype one was be set up in participants home to test stability, usability and integration with FOLD.
*Phase two:* Evaluation of second stage prototype of NOCTURNAL. The key functionality to be tested was the implementation of music and the possibly light linked to respond to sensor data.
*Phase three:* Evaluation of stable NOCTURNAL system and full integration into the FOLD Telecare system.

**Figure 1:**
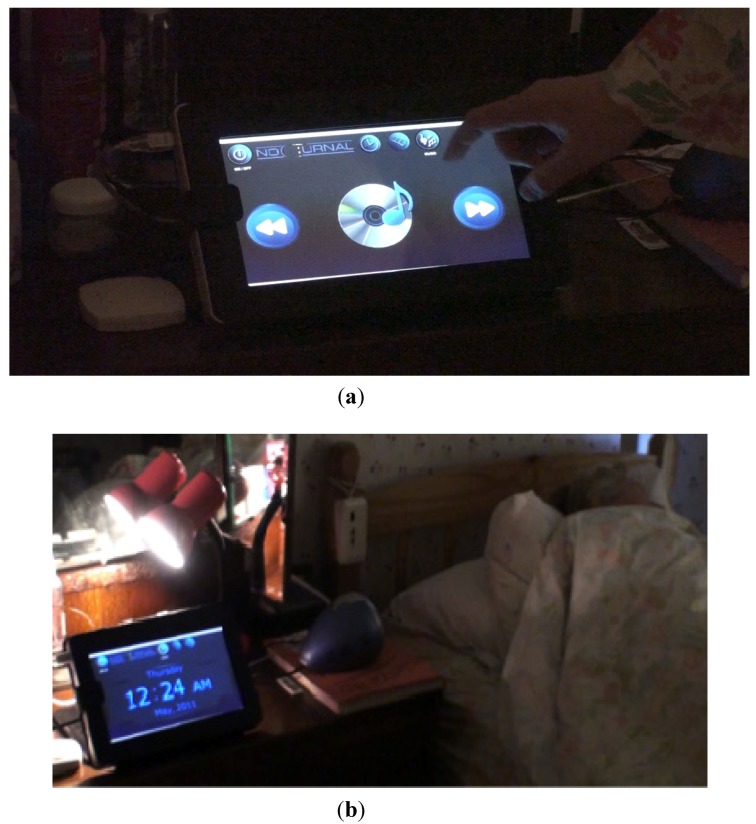
(**a**) Participant navigating through tablet to select personalized music; (**b**) Tablet PC on user’s bedside cabinet.

During this research we met face-to-face for qualitative interviews with the participants and their carers. The purpose of these interviews would vary dependent upon the stage in the research cycle. Initially we worked directly with people living with dementia to understand their real needs at home and then develop a system based on personalized preferences. The interviews would be scheduled at a time convenient for the participants and would last no longer than one hour. During these sessions the researcher would have prepared a guide on the topics to be covered. In the early phases this was based on understanding the lived experience of the participants, and then as the system was delivered into the home we explored what the participants thought about it and also asked them to demonstrate use. We would observe the participant at home, and ask them to work through some usage protocols. In addition we would show them the reports that were being generated by the system and explain what this information meant. The participants and carers would provide personal photographs, which would be uploaded onto the NOCTURNAL tablet shown in Figure 1 below. They and their carers also told us about their personal music choices and genres and this music was uploaded onto the tablet. At each stage of the system evolution we would revisit the people with dementia and explore how they are using the system, did they find it useful and was it stable for example. We met directly with informal carers who described the stress and challenges of supporting a loved one with dementia at home. All meetings with participants were held at a convenient time for them, often this was mid-evening. One to one interviews rather than in a focus group scenario for the people with dementia and their carers meant that over the lifetime of the project we got to know some participants and their families very well. In addition to this we planned a focus group with fit healthy older people to gain insight into their perspective on what we were proposing. 

The participants were included in all stages of the research cycle and offered the opportunity to take part in the 3 phases of iterative validation and evaluation of the NOCTURNAL platform. It was foreseen that each of these phases would result in a system being *in-situ* with the participant for up to 3 months to give meaning data of the system and user experience.

There were many research questions within this complex project, at a high level these could be stated as:

1. Has this NOCTURNAL technology the potential to be developed into the smart home platform to offer therapeutic intervention to people living at home with dementia?
2. Can people with dementia and their carers be directly involved in researc

## 3. Ethical Governance

This study gained ethical approval from the University of Ulster, Northern and Southeastern Health and Social Care Trusts, and ORECNI (08/NIR03/117). Aligned to this participant information sheets and forms to consent to take part in the project were prepared for both the person with dementia and their carer. Even after consent to join the research project a participant could change their mind at any time and withdraw from this work.

## 4. Findings

NOCTURNAL improved knowledge and understanding in many aspects of the technology design and service offerings. However critical insights have also been gained on user engagement—specifically for people with dementia. The findings will be presented under the categories of user specific, and technical results.

### 4.1. User Specific Findings

#### 4.1.1. Participant Recruitment

Participant recruitment was extremely challenging and remained live throughout the life span of the project. Having said that, one participant, who the case scenario below is based on, was recruited onto the project 21 October 2009 and remained with the research until the project closed January 2012. Many factors impacted on recruitment for example;

Appropriate referral for recruitment: Referrals for participants onto the project were sought from social work teams and medical staff from a local large mental health institution. Groundwork with the teams was essential to inform them about the project and inclusion criteria. The research team would hold information workshops with HSC staff (Social Workers and Psychiatrists) to inform them about the project and desire for suitable recruits. The consortium was then dependent on this team identifying suitable participants. We did ultimately achieve our target numbers, however:

○ This was a time intensive phase of the project.
○ Recruitment was very slow and regular contact with HSC staff was essential to remind them about our work.
○ A high number of participants were referred to the project team who were not suitable candidates.

There was a high attrition rate from the project as participant’s stage of dementia was often more established than initially thought. It was not uncommon for a participant to come onto the project, have the NOCTURNAL platform installed and then for the participant to withdraw from the project.

As a summary a total of *N* = 8 individual participants with dementia completed the 3 month final evaluation phase of the project with fully deployed systems. Significantly more people than eight were recruited and dropped out at various stages of the project. 

In addition to our work with participants who were living with dementia we also held a focus group with fit healthy older people once the second prototype was ready. We were interested to gain an understanding of elders who had no prior knowledge of the project and were not in the group of participants with dementia. This group provided really useful insight into their perspectives on what we were proposing. This group was facilitated by AGE NI and brought together 12 people of mixed gender who spent a morning with the researchers. During this focus group PowerPoint slides were used to explain about the project and the expectations of the session. A demo f the technical hardware and software was available for the group to touch and explore. At the end we passed around a non-standardised questionnaire for completion asking questions on a range of issues for example what type of services people would like to access, did they find this technology acceptable *etc*.

#### 4.1.2. User Acceptance of Technology Installation

Key themes identified from our work include:

- Promoting independence
- Maintaining dignity
- Maximising social inclusion
- Managing risk
- Providing stimulation

Face to face interviews enabled the team to explore aspects of user acceptance with participants in addition to talking with carers. These interviews took place at the participants’ homes and the carer could be present if the person wanted or it was considered beneficial. The approach was to develop short semi structured interviews to tease out aspect of design that would inform the next iteration. Most participants accepted the technology with no major problems. Participants liked the mobile component of the system and found it easy to navigate within. However some participants who stayed at home on the day of the installation did try to remove the system during the initial settling phase. One lady never settled on the bed sensor, and kept removing this. The system has to be removed in this case. 

#### 4.1.3. Carers Concerns

At initial interviews carers often reported that they were spending increasing amounts of time in direct contact with the person with dementia. They reported high anxiety about nighttime care as they often suspected that their relative was getting up at night. Often this was easily verified but sometimes carers only had anecdotal evidence for this or reports from neighbors. Either way this was affecting sleep of both person with dementia and their carer. 

#### 4.1.4. A Short Case Scenario

Mrs B lives alone in a rural area of Northern Ireland. She has early onset dementia, and a previous road traffic accident caused significant trauma to her lower limbs leaving residual motor problems, which impacted on her walking ability. She is aware of her diagnosis and frustrated by the deterioration in her memory. Her relationships with her family are tense at times as she feels she irritates them by constantly forgetting things. She also reports a decrease in her functional ability. Mrs B lives in social housing, a three bedroomed two-story home at the end of a terrace. Downstairs there is one reception room and a large kitchen/sitting room. There is a front garden and an enclosed rear paved area. When joining the NOCTURNAL project Mrs B was sleeping upstairs.

Mrs B had adopted numerous strategies to help with her many problems. She used notebooks and calendars and tried to keep develop a fairly standard approach to each day. During the one-to-one with the researcher Mrs B disclosed that she was concerned that someone was coming into her home at night. Each evening she left the kitchen tidy, however in the morning she would find crumbs and cups suggesting that someone had been in have food and a drink. She had no memory of her nighttime activity. She also didn’t want to share this information with her family as she thought it would cause them stress and they would also think she was leaving the door open to let strangers in. 

Using the nocturnal system we were able to clearly show Mrs B her nighttime activity and indeed clearly demonstrate the evenings when she was in fact coming downstairs and into the kitchen. The impact of this was very positive and reassuring for her; so much so she that told her relatives about this. In addition after discussing the risks of stair mobility in the dark at night Mrs B decided to move her bedroom downstairs the downstairs bathroom thereby removing the need to go upstairs.

The tablet PC of the nocturnal system was personalized to include music and family photographs. The family enjoyed putting this together and found it great fun to get the pictures together. Mrs B interacted well with the system during the day, in addition she liked the home screen with the day/date/time information.

When visiting a few months later Mrs B reported that she felt her condition was deteriorating. She was aware of the urge to go out of doors alone. She had awareness that this was potentially dangerous, and had one previous episode that gave her a fright. She described how this urge to leave the house would build in intensity from Sunday to Wednesday. The NOCTURNAL system was then adjusted to monitor closely the activity towards the doors. It was visible how an increase in activity was emerging. The system would then “kick in” a personal call from the monitoring station to talk to Mrs B and the monitor would dissuade from going out through the door. In addition the family accompanied Mrs B walking out doors and provided more opportunity for this when she felt the urge to outdoors.

Mrs B reported that knowing NOCTURNAL was there provided her with great comfort. She said it helped her relax and feel safe. The family reported similar feelings. The grandchildren were also involved, as they loved the technology of the touch screen home hub.

### 4.2. Technical Platform

**Figure 2:**
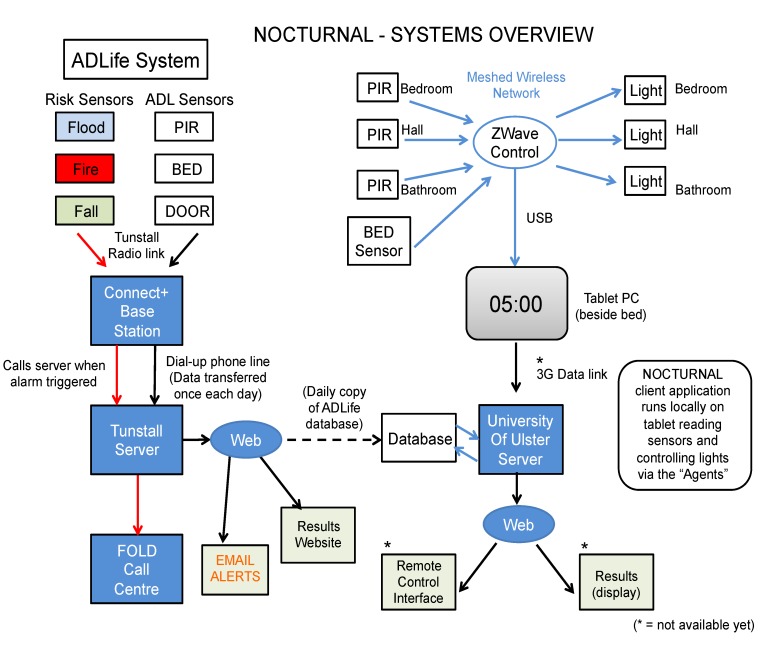
Overview of nocturnal system.

The technical architecture proposed to support NOCTURNAL is shown in Figure 2 below. It was decided to utilize commercially available hardware [39]; sensors, communication protocols, and a tablet PC and tailor the system using bespoke software, using an agent based approach for flexibility and extensibility. At the outset researchers concluded that the use of cameras to record or monitor images presented too many intrusive ethical concerns, and hence this source of information was not used. Components of the NOCTURNAL system were designed to integrate with the current care monitoring system (ADLife, from Tunstall, Figure 2 (left)). ADLife provides flood, fire and fall sensors, which are monitored remotely by care center staff to mitigate against serious risk and hazards. Additional passive infra-red (PIR) sensors, bed sensors and door sensors can be added to provide “context”, *i.e.*, where the care for person is located and give insight into their activities of daily living (ADL). This system represents the current “state of the art” in supported living, but requires active monitoring and person based intervention, e.g., the call center will telephone the monitored person should an alert be suspected and escalate support as required. 

The NOCTURNAL system (Figure 2 (right)) uses data from the home to monitor the participant during their routine daily activities and to intervene on their behalf using software based rules, which can then trigger actuators to provide lighting guidance, for example. Of course the knowledge to design the rules has to gleaned from experience in daily living. This knowledge was extracted from the ADLife database, which built up a profile of how sensors represented participant’s typical behavior over a period of weeks. NOCTURNAL focuses on assistance during hours of darkness and employs PIR sensors to locate the participant in the various rooms of the home and a bed sensor to detect whether he/she is in bed. The information is communicated wirelessly to a central hub, which for aesthetic purposes is a bedside table PC. The software runs locally on the tablet PC, interpreting the information from the sensors and triggering lights in sequence (for navigation purposes), or providing soothing therapy. For example if the participant gets up during the night to use the bathroom, lighting guidance is provided. The sensor and actuation events are store on a local database for further analysis. NOCTURNAL the following communication networks and protocols are used:

- Home networks, providing either wired and wireless connectivity with specialist protocols such as X10 used to supplement standard 802.x and z-wave networks;
- Wider connectivity to remote stakeholders (cares, monitors and healthcare professionals) via secure broadband services; and/or cellular services (GPRS, 3G) facilitating a sort of “virtual presence” for family members and occasional carers [40,41].

This picture below shows the NOCTURNAL tablet on the bedside cabinet of a service user. The home page functions as a reality orientation support tool providing information on date, time location *etc*., but the device provides additional functionality to support the playing of music and offering reminiscing therapy using pictures. Due to the wireless connectivity, the participant can easily move the tablet PC from room to room. 

The music can be personalised on set up and navigation to the music selection is straightforward This is shown in Figure 1a above. The researchers worked closely with each participant and their carers to elicit information on personal preferences for music. These were varied; one lady wanted religious, soft music and another had a surprising fondness for heavy rock. 

#### 4.2.1. Examples of Service Application

*Example 1*: if a participant is in bed the sensor will monitor movement on the mattress. If more than “normal” movement is detected the system will initiate (starting softly), the personal music choices, which will play until the person settles. The software uses empirical rules to determine the scope of normal movement, which of course will be different for each individual. This is an automated process. If the person doesn’t settle and this behavior is noted a notification to the care provider will be generated. Personalized photographs can accompany the soothing music. Direct interaction with the tablet was noted as a measure of usability.

*Example 2*: If the person doesn’t settle but gets out of bed, the light in the bathroom initiates to act as a guide and reduce tripping risk for the older person. If the person returns to bed from the bathroom the light dims, and the music gradually fades out after a set period of time.

#### 4.2.2. Visualisation of Ambient Assisted Living Service Data

The primary users of AAL services are the care recipients, but there are other important stakeholders including on-site formal carers, remote carers or monitors of AAL services, informal carers (including family, neighbors and friends) and those in charge of maintaining technical service provision. The task of interpreting the meaning in the data relating to the wellbeing and health of the care recipient has become complex due to the range of users and their overlapping roles in service provision.

There is a need for the AAL services to communicate vitally important information in an easy-to-understand manner to all users while maintaining the privacy of the care recipient and their informal carers. The manner in which these AAL services communicate information draws on visualization techniques. The visualization of AAL data is accessible on computer interfaces after authenticated access, but different modalities of access are also possible. For example, alerts can be “pushed” to mobile clients as the alerts are triggered. The AAL services generate data, which is then abstracted before being made available for visualization in decision support. The three main decision support modes are: communication of advice, provision of information, and management of (nuances of) alarm escalation. 

As an example within NOCTURNAL the following rules were applied to detect the sleep events:

(1) *Bed occupancy:*
  - *If (the bed sensor is “in” AND NOT (“out” in less than 1 min)) AND (no other PIR activated), then the bed entry is valid;*
  - *If (the bed sensor is “out”AND NOT(“in” in less than a minute)) AND (other PIR activated), then the bed out is valid;*
(2) *Sleep epoch: If the interval between each sleep epoch is less than 2 min, then merge the two epochs*

Several informative types of visual feedback on client’s daily, weekly and monthly sleep information and profiles were provided, including:

(1) Daily sleep pattern which displays a client’s daily sleep—wake cycles;
(2) Weekly sleep pattern which includes:
  - Client’s daily sleep pattern over past seven days. This section enables convenient visual comparison of sleep patterns and detection of unusual days;
  - Summary and trend of seven days of daily sleep-wake episodes; and
  - Summary and trend of seven days daily amount of sleep time.
(3) Monthly sleep pattern, which includes:
  - Summary and trend of four weeks of daily sleep-wake episodes; and
  - Summary and trend of four weeks daily amount of sleep time.

This visual feedback reflects the quantity, quality and rhythm of the daily sleep pattern, and data can be selected over a chosen period of time. Three main functions are implemented to view: sleep patterns, sleep hours and sleep episodes on daily, weekly and monthly basis. These visual reports were shown to carers and formal care staff to in one-one sessions to explore if they found the reports useful and easy to understand. Healthcare professionals can review clients’ profile and compare the changes of trend over various period of time, which may be helpful in determine the cognitive impairment stages [42]. 

To keep a consistent design theme different background colors are used, blue for daily information, green for weekly information and pink for monthly information. This information gleaned and the visualization reports were shared with family members who fulfilled the role of main carer. All found them extremely helpful and reassuring.

## 5. Discussion

Involving the end-users of technology in the development, validation and evaluation of device and services is essential. This is essential if wider uptake of the system, and continued use by target population is to be achieved. Government and non-government agencies representing the interests of people with dementia promote engagement directly with this group in research. The research literature confirms that research directed towards people with dementia, their carers and technology often experiences set-backs when migrating from the laboratory to the home [43]. Furthermore sample sizes are often small, and attrition rates high. This requires the research team to demonstrate considerable tenacity and skill to achieve successful deployment. The experience of the NOCTURNAL team completely concurs with these findings and challenges.

Robust ethical governance is an essential foundation for all research directly involving human participants. When vulnerable people, or those with reduced cognitive capacity are involved this is crucial. NOCTURNAL was scrutinized by four separate ethical review panels; the University of Ulster Research Ethics Committee, two Health and Social Care Trusts (Northern Trust and South Eastern) within Northern Health and the regional regulator for ethics (ORECNI). Ethical issues are not insurmountable and can be teased out, and navigated within [44,45]. The challenge with this type of user informed technology development is that by its very nature we want to seek the views of those who will use the device and service to help design, validate and evaluate it [46,47]. However, this creates a problem when seeking ethical approval, as the committees want to know exactly what the intervention (in this case technology device and service) will look like and what the participant experience will be. Gaining ethical approval to work with this group of older people with dementia on a technology-focused project was complex and challenging. This is completely understandable due to the vulnerable nature of the participants and the intrusion of technology into their homes. This required iteration of documentation and formal submissions to the various committees before a successful review was achieved. This added a significant time delay to the project time line of activity, as it was not possible to engage with any of the organizations above until ethical approval was given. For the consortium it was indeed a successful result in the overall activity of our work to gain successful ethical approval. One of the major challenges when involved in participatory research is that at the essence of the project is the desire for those who will use the system and services to help in the design. This is at odds with gaining ethical review as panels want to know what exactly you will present and show to participants. The iterative emergent nature of participatory research doesn’t neatly comply! A recommendation to others engaging in such research is to mitigate for a minimum three month period for a successful ethical outcome.

NOCTURNAL developed a multi-agent based platform, implemented in Jade and has three main roles: detect situations of interest with help of the sensing platform, decide whether the situation requires intervention, and delivering/follow up actuations to assess if the situation has improved. People with dementia and their carers were involved in the design and validation of the platform. At completion of the project a first generation prototype was developed. The next stage of work requires a more empirical research study using traditional methods to explore if indeed the technology can be used to provide therapy and reminiscence for people with dementia and impact on their experience as a valid healthcare intervention.

However as with many devices it is conceivable that it may not be perceived to be useful and hence will not be used. The factors influencing the adoption and (non)-abandonment of technologies are multifaceted involving the device, the services the user and the match between the two Whilst it might be hard to achieve user engagement with the mainstream population the task becomes more daunting when attempting to engage people with disabilities [48], and particularly those with dementia and their carers. This emerging issue it must be stressed was not directly attributed to either group, who our team found incredibly keen to be involved in this project. 

There are useful extensions that could further facilitate data analysis and system deployment and maintenance. The NOCTURNAL architecture (Figure 2) envisages that the data may be uploaded securely to a remote server (denoted UUJ Server) using a cellular network (e.g., 3G) for example, for further analysis, display and visualization. In addition this communication facility provides the opportunity to update and configure the software remotely. As far as the authors are aware, NOCTURNAL was unique in not only focusing on the nighttime needs of people with dementia but also working directly into the participant’s home. There were protocols in place by the research team to support this, for example as standard we deployed two people to be present at the participant’s home. This was developed to safeguard both the vulnerable participant and the researcher. Technology installations provided key learning not only in how to manage in a variety of homes, but also how to plan an install to best meet the needs of the person with dementia. 

Regarding the research questions posed we consider this project did take a significant step forward in terms of smart home technology for people with dementia by not only focusing on the particular needs of this group at night but also be developing technologies that had therapeutic potential *i.e.*, music and photographs both personalized which could be automated and activated by the sensors or used directly with carers. In this paper we present the caring technical protocols developed directly as a result of user engagement with the technology, based on real life scenarios. Our experience in this project is that people with dementia and their carers are very keen to be involved in research. They were motivated, interested and dedicated to the project. The main challenge was how to engage the right people and hence avoid unnecessary activity. This project took part in a significant geographical portion of Northern Ireland so logistically it was tricky to visit people easily without significant prior planning and specific time allocated.

The high attrition rates and the need to keep participant recruitment open for longer than initially envisaged was a challenge; however it is a reflection of the fact that most participants referred to the team had a more established dementia and were experiencing significant cognitive and functional problems as a result of this. 

## 6. Conclusions

This study addressed whether a participatory design involving people with dementia and their carers could utilize the advances in AAL to support people with dementia at nighttime period. It documented the system developed and validated in their home environments. Whilst undoubtedly there is further research to be done in this area, given the ageing population, NOCTURNAL demonstrated that robust solutions that rank highly with the users’ preferred needs can be deployed, accepted and used. Persistent waking during the night of a person with dementia poses a problem to all stakeholders. NOCTURNAL implemented a strategy which provided music as a therapy, lighting guidance for safety guidance, and used the existing ADLife system for backup. This work developed the comprehensive system to proof of concept stage. The most that can be said is that overall, user impressions of the systems were favorable. Further work however is required to explore this system as a healthcare intervention using traditional empirical research methods. 

AAL solutions are now available for real world deployment; sensors can be embedded in the environment relatively unobtrusively and tablets provide an aesthetically pleasing computation “home hub”, at minimal cost. Together with wireless protocols that can link these devices, the technology can no longer be seen as an obstacle. However real world deployment particular with this cohort continues to provide significant challenge.

NOCTURNAL aimed to bridge the gap in research to advance the understanding of how technology could be implemented to support the nighttime needs of people living with dementia. In addition, NOCTURNAL moved beyond the traditional perspectives of what services this might include and extended to develop a therapeutic element to the service. Nighttime presentation of dementia is well documented with the possibility of “sundowning” or wandering and the need to use the bathroom more often than with “just getting older”. All of these activities can potentially introduce of element of “risk” for the person with dementia. There is no doubt that technology has a strong and positive contribution to make to the challenges managing some of these risks. NOCTURNAL provided the opportunity to explore innovative service redesign and improve the quality of care for people living at home. The system is used as a way to increase independence with safety and to focus human interventions on those cases where it is really needed. The methods used during this project ensured that all key stakeholders (people with dementia, their families, healthcare providers and industry) were directly involved in the evolution of the system and service. This work contributes to advancing that understanding and also exploring beyond this, providing therapy and reminiscing in an ambient mode to orientate and comfort the individual. However the key issue often overlooked is; if technology is (part) of the answer, then what is the question or clinical problem to be solved? The only way to truly grasp this is to directly engage with the people who will use the devices and services.
